# Phylogenomic analysis expands the known repertoire of single-stranded DNA viruses in benthic zones of the South Indian Ocean

**DOI:** 10.1093/ismeco/ycae065

**Published:** 2024-05-01

**Authors:** Oliver K I Bezuidt, Thulani P Makhalanyane

**Affiliations:** DSI/NRF South African Research Chair in Marine Microbiomics, Department of Biochemistry, Genetics and Microbiology, microbiome@UP, University of Pretoria, Pretoria, 0028, South Africa; Department of Microbiology, Faculty of Science, Stellenbosch University, Stellenbosch 7600, South Africa; Department of Microbiology, Faculty of Science, Stellenbosch University, Stellenbosch 7600, South Africa; Centre for Epidemic Response and Innovation, The School for Data Science and Computational Thinking, Stellenbosch University, Stellenbosch 7600, South Africa

**Keywords:** biogeochemical cycling, CRESS-DNA, single stranded DNA viruses, Rep and Capsid proteins, South Indian Ocean, viral diversity

## Abstract

Single-stranded (ss) DNA viruses are ubiquitous and constitute some of the most diverse entities on Earth. Most studies have focused on ssDNA viruses from terrestrial environments resulting in a significant deficit in benthic ecosystems including aphotic zones of the South Indian Ocean (SIO). Here, we assess the diversity and phylogeny of ssDNA in deep waters of the SIO using a combination of established viral taxonomy tools and a Hidden Markov Model based approach. Replication initiator protein-associated (Rep) phylogenetic reconstruction and sequence similarity networks were used to show that the SIO hosts divergent and as yet unknown circular Rep-encoding ssDNA viruses. Several sequences appear to represent entirely novel families, expanding the repertoire of known ssDNA viruses. Results suggest that a small proportion of these viruses may be circular genetic elements, which may strongly influence the diversity of both eukaryotes and prokaryotes in the SIO. Taken together, our data show that the SIO harbours a diverse assortment of previously unknown ssDNA viruses. Due to their potential to infect a variety of hosts, these viruses may be crucial for marine nutrient recycling through their influence of the biological carbon pump.

## Introduction

Disentangling the phylogenetically diverse assemblages of bacteria, fungi, phytoplankton, and viruses and their contributions to ecosystem services in the global ocean remains a major endeavour. There is strong evidence that these assemblages influence the biological carbon pump in marine environments [1, 2]. Among these assemblages, viruses are the most numerically abundant [3], and recent evidence has demonstrated their profound influence on prokaryotic lifestyles in the oceans [4–8]. These studies have shown that viruses play key roles in determining ecological patterns in marine ecosystems and mediating nutrient recycling through the transfer of genes between both eukaryotic and prokaryotic hosts [9–11]. Through lysogeny, viruses enhance the release of organic matter, promoting the recycling of nutrients such as dissolved organic carbon, through the microbial loop [12]. While there are increased insights regarding viral contributions, most studies have focused on limited geographic locations. As a result, comparatively less is known regarding the phylogeny and function of single- and double-stranded DNA viruses in large regions of the global ocean.

An accumulating body of research suggests that viruses are central drivers of metabolic processes through auxiliary metabolic genes (AMGs) [13–15]. Previous studies have also shown that double stranded DNA (dsDNA) viruses mediate key metabolic processes, known to modulate microbial metabolic pathways during infection [16–19]. Most studies on viral diversity and functional contributions have been derived from studies conducted mostly in euphotic oceanic zones [11, 20–23]. This has resulted in a substantial knowledge gap regarding the evolutionary structure and function of viruses in benthic ecosystems. Current studies on benthic ecosystems have, however, provided several insights regarding the diverse dsDNA prokaryotic viruses and their encoded AMGs [24–28], and less on assemblages and potential functional contributions of ssDNA viruses.

More recently, uncharacterized groups of ssDNA viruses associated with vertebrates from terrestrial environments have become the subject of extensive research [29–34]**.** Although these studies have provided substantial insights regarding the diversity and versatility of ssDNA, the results have led to an overrepresentation of virus sequences from several habitats including terrestrial ecosystems and mammal derived samples [35–37]. Several studies have noted the lower proportion of studies on ssDNA viruses in marine ecosystems [36, 38, 39]. Given the importance of viruses as mediators of the biological carbon pump, the lack of studies on ssDNA viruses may limit our understanding regarding their functional contributions. The diversity and functional contributions of ssDNA in the oceans may substantially contribute to marine nutrient recycling and the biological carbon pump.

Previous studies on viral communities in benthic zones have demonstrated that ssDNA viruses constitute dominant constituents of these environments [40, 41]. However, to the best of our knowledge, the evolutionary relationships, diversity, and distribution of ssDNA viruses in the SIO remains unexplored. We predict that the distinct environmental conditions and water masses in the SIO may select for phylogenetically diverse ssDNA viruses. Here, we explore the diversity and phylogeny of ssDNA from benthic zones of the SIO. In addition to using conventional approaches to search for putative viral contigs, we applied a hidden Markov model (HMM) workflow to expand the current repertoire of ssDNA viruses.

## Materials and methods

### Sample collection and molecular ecological analyses

Seawater samples were collected aboard the *RV SA Agulhas II* from the Crossroads transect in the South Indian and Southern Oceans as detailed previously [42, 43]. As part of the cruise, 39 samples (13 × 3) were collected across a 1000 km transect. At each site, three samples were collected from the deepest depth (Supplementary Table S1). A Sea-Bird SBE-911plus V2 CTD System (Sea-Bird Electronics, Inc., Bellevue, Washington, USA) was used to collect samples ~10 m above the seafloor. Depending on the conditions, the CTD was retrieved within 5 hours. Once on deck, 5 L of seawater was retrieved from each of the three Niskin bottles and subjected to a two-step filtration process to allow for the collection of particle-associated viruses (hereinafter referred to as viruses), potential symbionts including candidate phyla radiation, and free-living microbial biomass. Following filtration, all samples were immediately stored at −25°C until processing.

DNA was extracted from membrane filters using a phenol–chloroform method [44] with extractions performed in duplicate, with minor modifications. Specifically, the pH of the extraction buffer was adjusted from 8 to 9.5 to compensate for low DNA concentration. Using sterile forceps, both 0.45 and 0.2 μm membrane filters were cut in half and used as the samples in the protocol, and as a result the number of glass beads used in the protocol was lowered from 0.4–0.5 to 0.25 ml (0.10–0.11 mm diameter). Samples with the highest DNA concentration (*n* = 6) were sent for sequencing at the Molecular Research DNA (MR DNA) sequencing facility (Shallowater, TX, USA). These six samples are pseudo replicates from same sampling sites as indicated in SupplementaryTable S1 (CR11, CR12 for CR1; CR21, CR22 for CR9; and CR31, CR32 for CR10). Due to generally low DNA yields, whole metagenome amplification was performed using REPLI-g Midi kit (Qiagen, Hilden, Germany). Libraries were prepared using the Nextera DNA Sample preparation kit (Illumina, Inc., San Diego, CA) with a small insert size (<1 kbp). The final libraries were then pooled and sequenced as paired end reads for 300 cycles, using the Illumina HiSeq 2500 system (Illumina, Inc. San Diego, CA, USA)**.**

### Bioinformatics analysis

#### Shotgun metagenome analysis and processing

Raw metagenomic reads were inspected for quality, and the presence of sequencing adapters, using FastQC (www.bioinformatics.babraham.ac.uk/projects/fastqc). Following this, the reads were processed using BBsplit version 38.00 to remove PhiX174 (CP004084.1) using default settings. The resultant PhiX174 free reads were further trimmed using Trimmomatic version 0.36 [45]. The total sequencing coverage was estimated using Nonpareil version 3.301 [46] (Supplementary Fig. S1). We assembled the metagenomes using metaSPAdes version 3.14.1 as detailed previously [47] following default parameters. All contigs <1000 bp were removed and the remaining sequences were used for downstream analyses.

#### Bacterial taxonomic classification and functional annotation

To estimate the distribution and taxonomy of the taxa recovered from the metagenomes, contigs were aligned against a non-redundant (NR) protein database from the National Center for Biotechnology Information (NCBI) database [48]. We used DIAMOND BLASTx version 2.02.2 with the default settings [49]. The outputs were analysed using MEGAN version 6.20.19 [50] to estimate taxonomic distributions and determine overrepresented taxa across all metagenomes. Contigs for the top five most represented taxa were extracted and analysed using Kofamscan version 1.3.0 to determine their metabolic potential with the default settings [51].

#### Viral prediction and quality processing

To assess the distribution and classification, two approaches (detailed below) were used to predict viruses. For the first approach, we used standard viral prediction pipelines, whereas the second relied on the use of a HMM based approach [52].

**Approach 1**: All six assembled metagenomes were used to search for putative viral contigs using a combination of Virsorter version 2.2.3 [53] and VirFinder version 1.1 [54]. Contigs with sizes ≥1 kb, predicted using both Virsorter2 version 3 SOP (dx.doi.org/10.17504/protocols.io.bwm5pc86), as well as those predicted to have *P*-value <.01 by VirFinder, were retained and merged for downstream analyses. These merged contigs were clustered into viral operational taxonomic units (vOTUs) using CD-HIT version 4.8.1 [55], based on 95% sequence identity >80% of the shortest contig. The resultant vOTUs contig were processed using CheckV version 0.70 [56] to estimate overall quality and completeness (Supplementary Tables S2 and S3). Putative viral contigs, that were designated as complete, high, medium, and low were retained. These contigs were post-processed using the geNomad pipeline version 1.6.1 [57] with the default parameters. Contigs that were classified as affiliated with the class *Caudoviricetes* and order *Petitvirales* were assessed for the presence of auxiliary metabolic genes using VIBRANT version 1.2.1 [58], with the default specifications. The lifecycles associated with these putative viruses were determined using BACPHLIP [59] with the default settings. Lytic/virulent phages were identified with a minimum lifecycle prediction score of ≥0.8. Following this, we inspected a set of putative ssDNA-specific viruses. Contigs (≥1 kb) predicted as ssDNA viruses by Virsorter version 2.2.3 were retained. These contigs were further clustered into viral OTUs using CD-HIT version 4.8.1 and further estimated for quality and completeness as detailed earlier. We retained contigs designated as complete, high, medium, and low quality. These putative virus predictions were validated using BLASTp [60] with e-value 1e-05, against the NR protein database from the NCBI (accessed January 2023) [61].

**Approach 2**: To generate HMM-based profiles, we retrieved all *Cressdnaviricota* and *Phixviricota* protein sequences from GenBank [61], (https://github.com/SAmicrobiomes/ssDNA). To reduce redundancy, these sequences were clustered at 95% amino acid identity >90% of the shortest sequence, using CD-HIT version 4.8.1 [55]. The resultant sequences were compared, using all-vs-all BLASTp with e-value 1e-5. The output sequences were further clustered, using Markov cluster (MCL) algorithm [62] with the inflation parameter set to 1.5. Clusters with proteins ≥10 annotated replication initiator (Rep) and major capsid (VP1) were aligned using MAFFT version 7.487 [63] with the —auto parameter. The alignments were used to create HMM profiles, using HMMER version 3.3.2 [52]. The profiles were searched against protein sequences, which were predicted using the -p meta function in Prodigal [64], from all metagenomes using HMMSCAN version 3.0 [65]. Metagenomic sequences, that shared similarity with conserved *Cressdnaviricota* replication initiator (Rep) and *Phixviricota* major capsid (VP1) proteins, with HMMSCAN scores ≥50 were retrieved for downstream analysis. Viral contigs, predicted to harbour Rep and VP1 proteins, were validated using BLASTp [60] with e-value 1e-05 against a non-redundant (NR) database acquired from the NCBI as well as HHpred [66] against the Protein Data Bank (PDB) and Protein Family Database (Pfam) database.

### Phylogenetic analysis of single stranded DNA viruses

Complete Rep and VP1 protein sequences, from both our metagenomes and GenBank viral datasets, were used to reconstruct phylogenies based on conserved amino acid sequences. Rep proteins, predicted from our metagenomes, were compared with those from ssDNA viruses and plasmid sequences from previous studies [67]. These protein sequences were aligned using MAFFT-linsi and trimmed using Trimal with gap threshold 0.15 [68]. Phylogenetic trees were computed using FastTree with options -spr 4 -mlacc 2 -slownni -lg [69].

## Results and discussion

### Diverse putative hosts dominated by prokaryotes

Viruses are crucial for the biological carbon pump and regulate microbial community structure and abundance, determining the genetic diversity and evolution of their hosts [70–74]. In turn, there is some evidence that marine viruses co-exist with potential hosts, which include phylogenetically diverse eukaryotes and prokaryotes [22, 71, 75–77]. For instance, previous studies have shown that eukaryotic hosts include copepods [78] and protists [79]. However, the diversity of potential hosts in understudied environments such as the SIO remains unknown.

**Figure 1:**
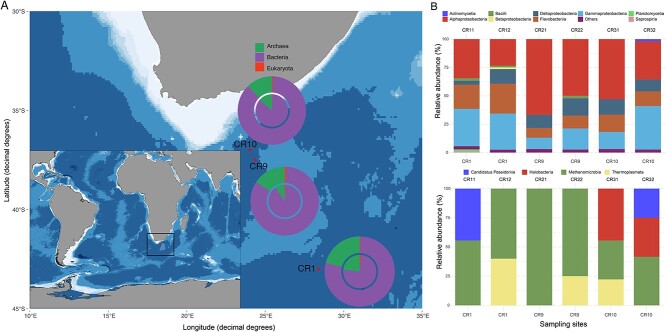
(A) A map showing the six sampling locations in the South Indian Ocean. The map also includes an overview of microbial diversity (pie charts) at each sampling location. (B) Bar plots showing the relative abundances o bacterial and archaeal classes, at each sampling site.

Our dataset provides an overview of the taxonomy of bacterial, archaeal, and eukaryotic hosts in benthic zones of the SIO. The data suggest that bacteria and archaea may be the dominant hosts, compared with eukaryotes which constituted a minor fraction of our sequences (Fig. 1A). This finding is consistent with previous studies, which have demonstrated that prokaryotes far outnumber eukaryotes in all ecosystems [4, 80–83]. Taxonomic classifications suggests that three bacterial (*Proteobacteria*, *Candidatus Marinimicrobia*, and *Bacteroidota*) and one archaeal (Thaumarchaeota) phyla were the most overrepresented in the benthic SIO (Fig. 1B). Other studies on the diversity and distribution of marine microbiota in aphotic zones have reported similar findings [84–86]. Some of these taxa constitute ecologically rare taxa, which may represent active free-living bathypelagic microbiota [87] and may disproportionately contribute to the sequestration of key nutrients.

Although sequences affiliated with Eukaryota were present at comparatively low abundances, several taxa including Chlorophyta, Streptophyta, Foraminifera, Ascomycota, and members of the ecologically diverse SAR supergroup were found (Supplementary Fig. S2). These taxa may have originated from deep-sea floor unicellular and multicellular species. We argue that some of these species may host eukaryotic ssDNA viruses found in our dataset. Our results are consistent with previous studies, which reported generally low abundances of eukaryotes in other benthic environments such as the Black Sea [88]. Based on their relatively high abundances, we predict that prokaryotes may be the primary hosts of marine viruses, which ultimately mediate important functional processes in the SIO.

### Widespread functional capacity in diverse prokaryotic hosts

**Figure 2:**
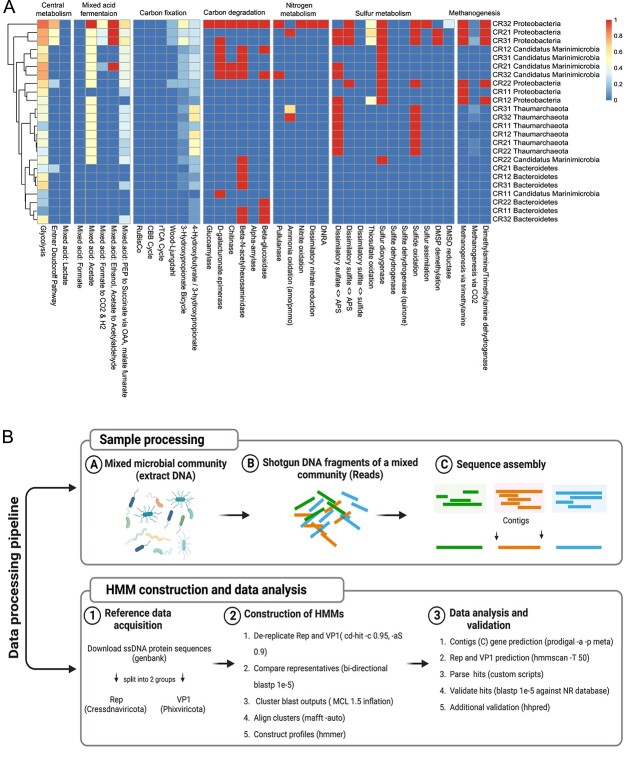
(A) Heatmap showing the metabolic potential of the four most overrepresented taxa, at each site. The plot shows the (B) overview of the methodological workflow followed in this study including sample processing and hidden Markov model (HMM) construction and data analysis.

To reduce the knowledge deficit regarding contributions to ecosystem services, we determined functional capacity and putative ecological contributions of bacteria and archaea in the SIO. Metabolic analysis revealed a suite of complete pathways linked to carbon degradation, nitrogen, methanogenesis, and sulphur recycling (Fig. 2A). In addition to providing the first such data for this region, the results are consistent with recent findings showing that deep oceans possess remarkably diverse capacity for functional processes [89–92]. Linking these functional genes to microorganisms suggests that some taxa may exclusively drive specific metabolic processes. For instance, Proteobacteria appear to be the only taxa with metabolic capacity for methanogenesis, suggesting that they may contribute to oxidizing methane in the deep environments [93, 94]. This result suggests that these numerically dominant taxa may mediate key ecosystem processes in the SIO. Results from functional analysis suggests that some metabolic roles may be driven by a consortia rather than one numerically abundant microorganism. For instance, genes for sulphur metabolism were found in sequences affiliated with Candidatus Marinimicrobia, Proteobacteria, and Thaumarchaeota. Among these, ammonia oxidizing Thaumarchaeota appear to be the only taxa, with a complete pathway for dissimilatory sulphate reduction and sulphide oxidation. This result suggests that these Thaumarchaeota may play especially important roles in sulphur recycling [95, 96]. This observation also suggests that, in addition to other groups associated with marine and terrestrial environments (i.e. *Euryarchaeota*, *Crenarchaeota*, and *Aigarchaeota*) [97], Thaumarchaeota may augment the metabolic contributions of candidate sulphate-reducing archaea. Collectively, the detection of these genes provides some indication of the metabolic capacity in aphotic SIO waters, which may influence important ecological processes.

### Evidence of extensive viral diversity and functional potential in the deep South Indian Ocean

Despite the importance of the SIO in modulating global climate and heat uptake in the ocean [1, 42, 98], we lack taxonomic insights regarding the functional roles of viruses. Using two of the most widely used pipelines [54, 99], we explored viral communities in deep SIO waters. Viral predictions were combined, checked for quality and completeness, and clustered into 3076 putative viral OTUs. In total, 1427 (46.4%) of these vOTUs were classified into known prokaryotic viral taxa. These viruses included members of the class *Caudoviricetes* (991), which included families *Straboviridae* (*n* = 3), *Kyanoviridae* (*n* = 2), *Autographiviridae* (*n* = 1), and *Herelleviridae* (*n* = 1). In addition, several sequences were affiliated with taxa from the order *Petitvirales* (436), including members of the *Microviridae* (*n* = 406) family.

Only a small fraction of the total vOTUs appear to harbour AMGs. From 62 vOTUs, we identified 78 AMGs (Supplementary Table S4). These AMGs were mostly associated with functional traits related to the synthesis of amino acids, carbohydrates, secondary metabolites as well as terpenoides/polyketides metabolic pathways (Supplementary Table S5). Several of these AMGs related to amino acid metabolism included 2OG-Fe(II) oxygenases (arginine and proline metabolism). Previous studies suggest that these AMGs may modulate host nitrogen metabolism, stress response, and DNA repair mechanisms [100–102]. Some of the genes linked to these AMGs are involved in the metabolism of cysteine and methionine, linked to carbon, nitrogen, and sulphur utilization, and the reprogramming of cells to anabolic states [103]. The presence of viral AMGs, previously implicated in sulphur metabolisms (i.e. *dcm*, *cys*H, and *met*K) [104], correlates with the most complete pathways in our datasets. These pathways were linked to the four most overrepresented phyla in our metagenomes. Genetic evidence suggests that viruses may contribute substantially to bacterial metabolic reprogramming in deep SIO waters [105]. Determining the lifecycles of Caudoviricetes found in the SIO suggests that these viruses may favour lytic (*n* = 948) over lysogenic (*n* = 14) cycles. This is consistent with previous reports of high prokaryotic mortality due to viral lysis in the dark ocean [106]. The study by Lara *et al.* [106] further suggests that a preference for viral lytic cycles may result in the production of dissolved organic carbon, supporting respiration in the dark ocean [107]. To estimate the diversity of our Caudoviricetes, we clustered all 991 representative contigs against viral RefSeq database (release 219) using 95% ANI across 85% of the sequence using a method detailed previously [56]. However, none of our contigs clustered with any of the viruses from the reference dataset, suggesting that the deep SIO hosts potentially novel dsDNA viruses.

To explore the diversity of eukaryotic DNA viruses in our dataset, a set of putative viral contigs highlighted as ssDNA by Virsorter 2.2.3 were retained [53]. These were clustered into 7627 viral OTUs. Of these, 6018 vOTUs were validly assigned and predicted to be complete (24), high (1559), medium (2146), and low (2289) quality. Sequence similarity searches, using these assigned contigs, were used to estimate the diversity of ssDNA viruses. A total of 3440 contigs, with blast descriptions containing the terms circo, CRESS, circular genetic, circular virus/DNA, and which either possessed or lacked replication initiator proteins, were classified as rep-encoding ssDNA viruses. Among these, 150 contigs were classified as *Microviridae*, in addition to 810 other contigs associated with dsDNA phages. These numbers suggest a high proportion of false positive predictions (Supplementary Table S6).

### Uncharacterized circular Rep-encoding ssDNA sequences dominate deep South Indian Ocean waters

Due to inherent methodological differences, current *in silico* approaches may yield contrasting results regarding viral divisions. While previous studies have revealed a diverse array of viruses [25, 108], sequence classification using standard approaches leads to false positive predictions of prokaryotic ssDNA viruses [109, 110]. A consequence of these false positive annotations may be the assignment of dsDNA viral contigs as ssDNA. To avoid this common issue, we used a Hidden Markov Model (HMM) based pipeline described previously [52] to investigate the diversity of Microviruses and Circular Rep-Encoding ssDNA (CRESS) viruses, in the SIO (Fig. 2B). Using this approach, we established HMM profiles for conserved Rep and VP1 proteins from Cressdnaviricota and Phixviricota viral sequences retrieved from GenBank [111]. Based on these profiles, HMM searches across all metagenomes, revealed 3260 and 344 putative Rep and VP1 complete protein sequences, respectively. To reduce redundancy, these sequences were subsequently clustered (at ≥95% amino acid identity, over 85% of the shortest sequence), resulting in 2307 (Rep) and 209 (VP1) representative protein sequences. The validation of these hallmark sequences, against a non-viral specific database such as NR, suggests that 2303 and 209 of Rep and VP1 sequences shared sequence similarities with both rep-encoding viruses and *Microviridae*, respectively. The remaining four Rep sequences, without hits against NR database, were further validated using HHpred against PDB and pfam databases (Tables S7 and S8).

**Figure 3:**
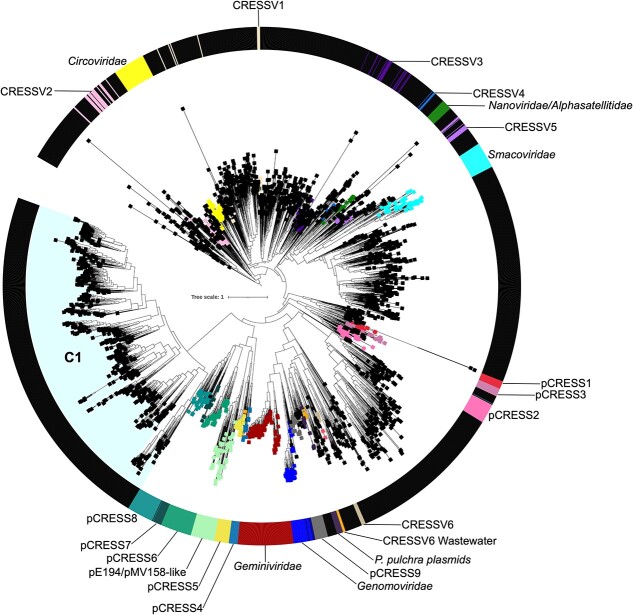
An unrooted maximum likelihood phylogenetic tree showing the diversity of Rep protein sequences. The black squares represent Rep proteins, from the South Indian Ocean, and CRESS DNA viruses. The other groups shown include rep proteins retrieved from a previous study by Kazlauskas *et al*. [67].

To reduce the knowledge deficit regarding these viruses, we estimated the diversity and phylogeny of CRESS sequences from the SIO. Phylogenetic analysis was used to compare 2307 Rep sequences with the 709 reported by Kazlauskas *et al*. [67] (Fig. 3). Our maximum likelihood reconstructions suggest that sequences from the SIO may represent unclassified CRESS. These sequences were predominantly assigned with categorical Groups 1–5 as well as *Circoviridae*, which are known to infect a wide range of hosts including mammals and diatoms [112–114]. Consistent with previous studies [41, 115], these viruses were highly abundant in our samples. It is possible that these viruses may interact with phylogenetically diverse eukaryotic hosts. These hosts may include members of the division Chlorophyta and Streptophyta, Foraminifera, Ascomycota and the prevalent SAR supergroup (Supplementary Fig. S2). Our phylogenetic analysis revealed distinct unclassified clades, including C1 which appears to be comprised exclusively by viruses from the SIO (Fig. 3). These distinct clades expand the known diversity of marine viruses. In addition, sequences from the SIO include clades representating several as yet unidentified rep-encoding CRESS viruses. The data strongly suggests that these viruses may have a broader host range preference than previously thought. In addition, the may the environmental conditions in these deep SIO waters may select for distinct CRESS viruses adapted to these harsh conditions. The analyses further revealed that one sequence clustered with PCRESS8 and three grouped with Genomoviruses which are known plasmids and fungal pathogens, respectively [116, 117].

**Figure 4:**
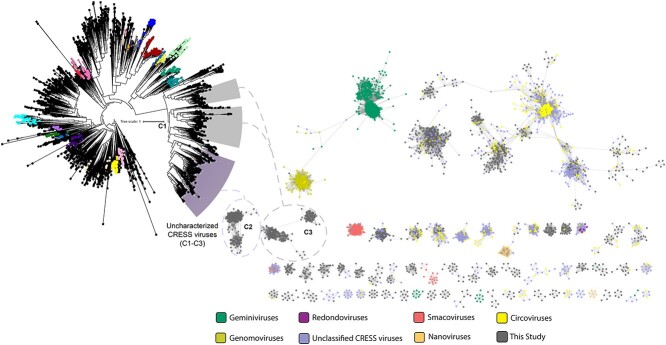
An unrooted maximum likelihood phylogenetic tree (A) and a sequence similarity network of rep protein sequences indicating some of the potentially novel CRESS viral families (B) from our South Indian Ocean data, annotated as C1 to C3.

To explore the diversity of ssDNA viruses 2307 (Rep) and 209 (VP1) non-redundant proteins, recovered in this study, were compared with GenBank Rep and VP1 protein sequences. These sequences were used to generate family-level clusters of sequence similarity networks (SSNs), as described previously by Kraberger *et al.* [118]. These SSNs included 87.1% Rep (2010) and 98.5% VP1 (206) of ssDNA protein sequences from the SIO dataset. Consistent with the phylogenetic analysis, SSN generated by comparisons of Cressdnaviricota Rep proteins showed that at least 89% (1792 of 2010) of the Rep sequences from our dataset, clustered with Circoviruses and unclassified CRESS viruses (Fig. 4). The SSNs also suggests that several sequences from our data represent distinct clusters, which were not similar to those deposited on GenBank. These divergent clusters (C2 and C3) overlapped with the distinct CRESS like viral C1 clade in the phylogenetic tree. In addition to confirming some overlap between the two methods, this result highlights the high diversity of ssDNA viruses in deep SIO waters (Fig. 4).

Contrary to CRESS signatures, HMM searches suggest that the numbers of Phixviricota-like VP1 protein sequences in the SIO may be low. Additionally, Phixviricota VP1-based SSN indicated that prokaryotic viral sequences from the SIO may be similar with known Microviruses (Supplementary Fig. S3). This result is somewhat surprising and is in contrast to previous studies, which have reported that Microviruses from benthic marine environments were highly abundant and diverse, compared with other viruses from the same family [119]. Based on our findings, we propose two possibilities. The first is that benthic Microviruses from the SIO may be less divergent due to the ecological selective pressures. Alternatively, the underrepresentation of these viruses in our data may be due to technical challenges. For instance, amplification biases during library preparation have been previously shown to obscure viral abundances in several studies [120, 121]. However, based on the remarkably low number of studies on the SIO, and the limited datasets, it is reasonable to conclude that these viruses may represent highly diverse and novel lineages. Validation of metagenomic assemblies supports this assertion and revealed that 2590 contigs from our dataset may be associated with circular genetic elements, which were previously described by Tisza *et al.* (2020) [34]. SSNs indicated that 12 and 4 clusters (of the total 161, with cluster sizes ≥ 5) were constituted by ssDNA viral Rep and Capsid associated proteins, respectively (Supplementary Fig. S4). This result suggest that the diversity of ssDNA viruses in the SIO may be as similar to levels previously reported in the North Atlantic Ocean [122] and other polar aquatic environments [123].

Taken together, the diverse ssDNA viruses identified in this study suggests that the relationships between virus and host populations may be dynamic or substantially driven by physicochemical properties in the deep SIO [33]. However, the functional potential of eukaryotic viruses in the environment remains under-explored. Our results showing high abundances of Cressdnaviricota suggests that these viruses contribute significantly to the biological carbon pump in the SIO. Alternately, based on the phylogenetic evidence from our study it is reasonable to predict that viruses may have profound effects on ecological processes across the water column, from the surface to the deepest depths. These effects may include recycling dissolved inorganic carbon in aphotic zones. However, studies deploying a marine snow catcher are required to confirm the role played by viruses in suspended and sinking particulate matter in the SIO. Given the importance of the SIO in global climate and nutrient circulation, these studies may shed light on the influence of these viruses on nutrient recycling of carbon and nitrogen.

## Supplementary Material

Figure_S1_ycae065

Figure_S2_ycae065

Figure_S3_ycae065

Figure_S4_ycae065

Supplementary_Tables_S1-S8_ycae065

Supplementary_information_for_ycae065

## Data Availability

Raw sequence data linked to study have been deposited to the NCBI SRA under accession number PRJNA894371. Supplemental materials are available on the following link: https://doi.org/10.6084/m9.figshare.24032697.
