# Supplementary figures and images for: Phylogenomic analysis expands the known repertoire of single-stranded DNA viruses in benthic zones of the South Indian Ocean
Source: ISME Commun. 2024 May 1;4(1):ycae065. doi: 10.1093/ismeco/ycae065 (PMC11128263; doi:10.1093/ismeco/ycae065)

# Nonpareil Curves

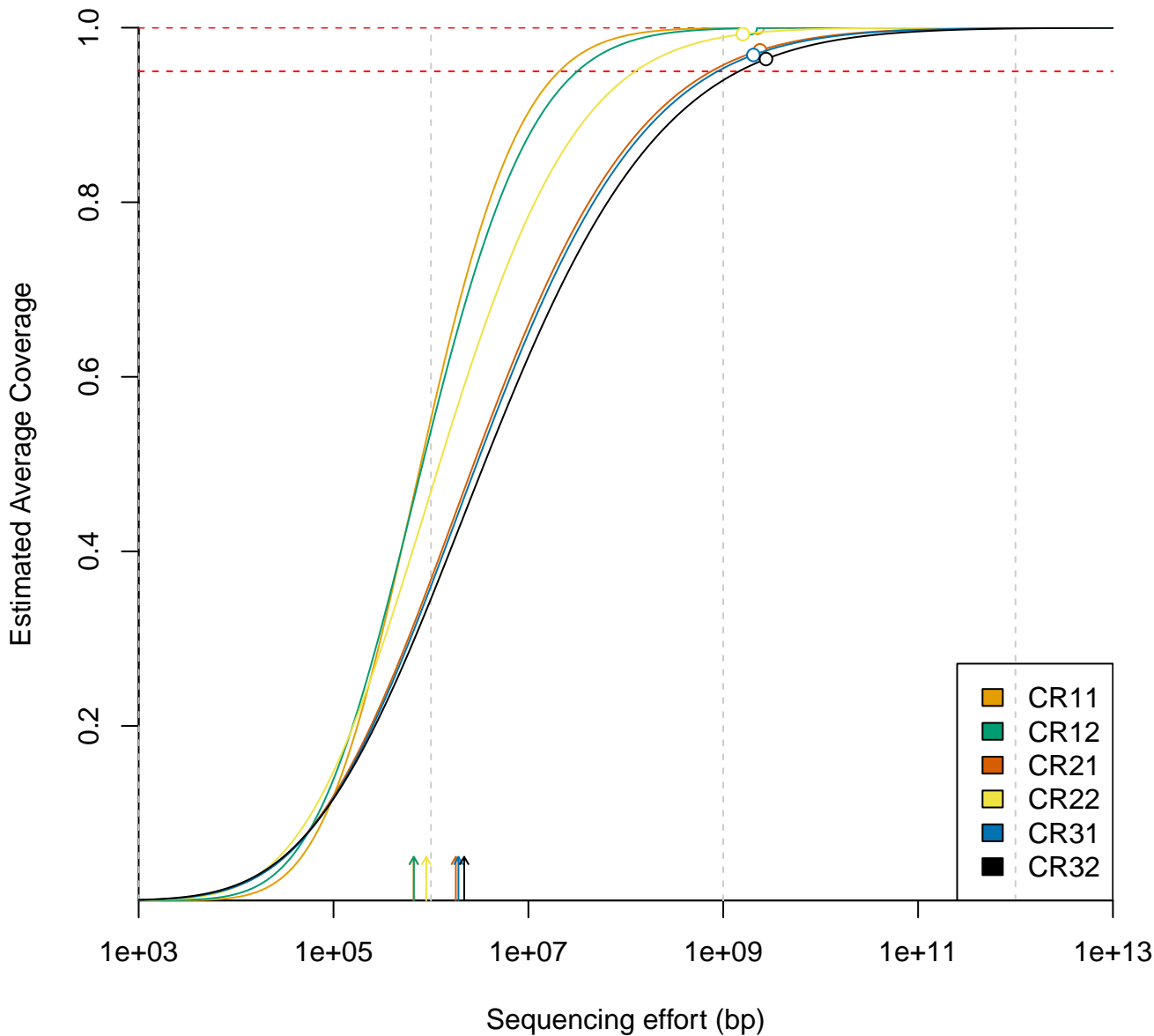

Supplement: Figure_S1_ycae065 [file figure_s1_ycae065.pdf]

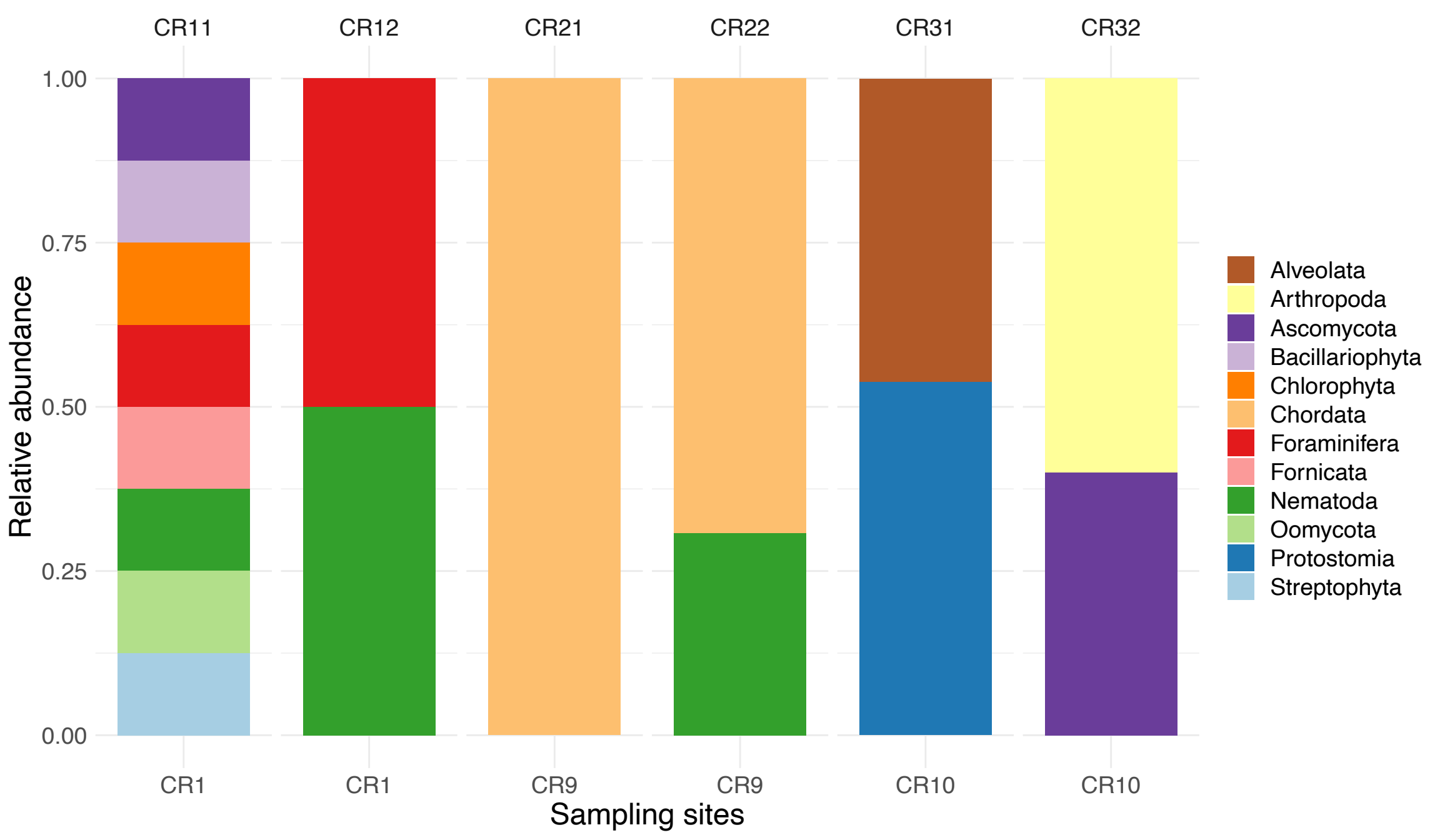

Supplement: Figure_S2_ycae065 [file figure_s2_ycae065.pdf]

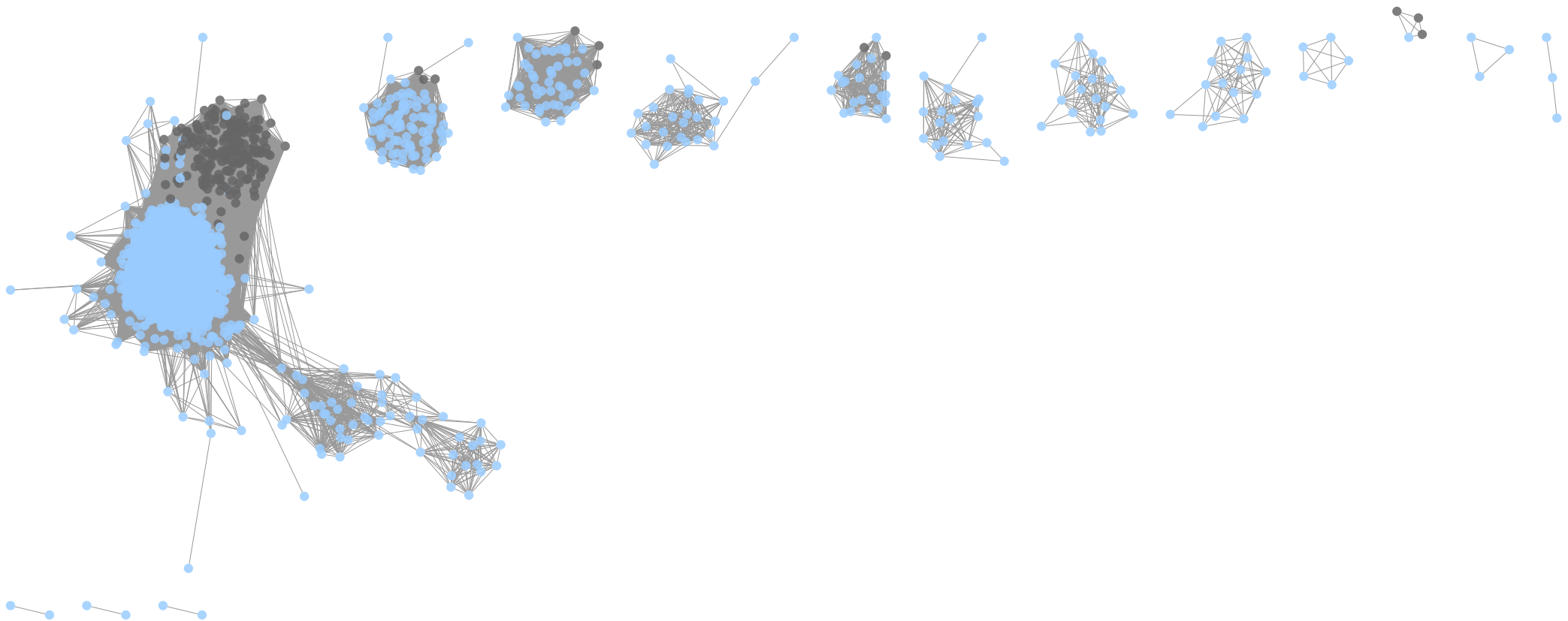

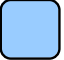 **Microviruses**

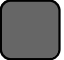 **This study**

Supplement: Figure_S3_ycae065 [file figure_s3_ycae065.pdf]

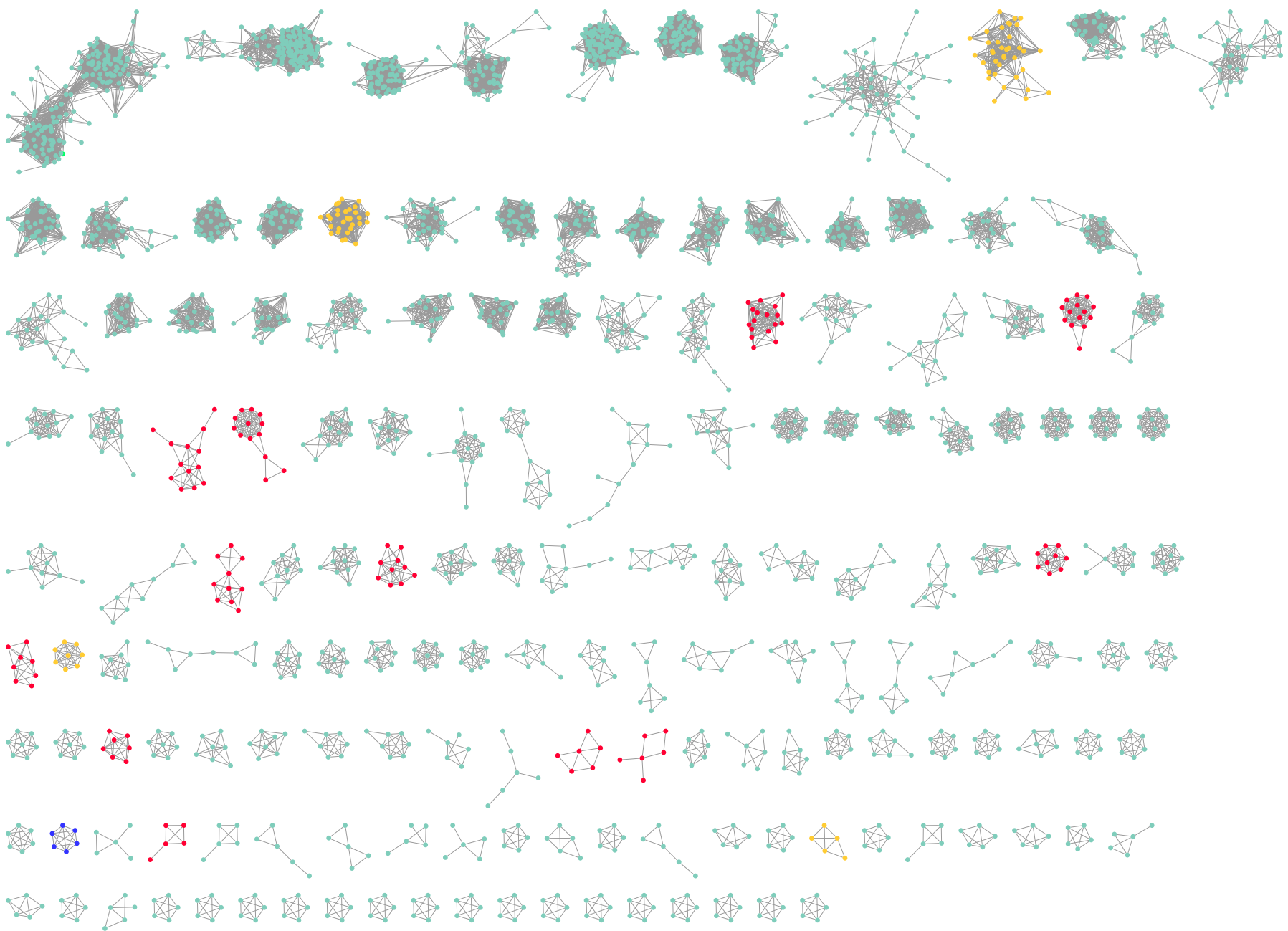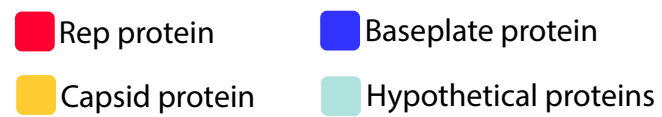

Supplement: Figure_S4_ycae065 [file figure_s4_ycae065.pdf]
