# Supplementary material for: Phylogenomic analysis expands the known repertoire of single-stranded DNA viruses in benthic zones of the South Indian Ocean
Source: ISME Commun. 2024 May 1;4(1):ycae065. doi: 10.1093/ismeco/ycae065 (PMC11128263; doi:10.1093/ismeco/ycae065)
Supplement: Supplementary_information_for_ycae065 [file supplementary_information_for_ycae065.docx]

**Supplementary information for**

**Phylogenomic analysis expands the known repertoire of single-stranded DNA viruses in benthic zones of the South Indian Ocean**

Oliver K.I. Bezuidt^1,2*^and Thulani P. Makhalanyane^2,3 #^

**Affiliations:**

1. DSI/NRF South African Research Chair in Marine Microbiomics, Department of Biochemistry, Genetics and Microbiology, microbiome@UP, University of Pretoria, Pretoria, 0028, South Africa
2. Department of Microbiology, Faculty of Science, Stellenbosch University, Stellenbosch, 7600, South Africa
3. The School for Data Science and Computational Thinking, Stellenbosch University, Stellenbosch, 7600, South Africa

**Included:**

Supplementary table legends (included in Excel file)

Supplementary figure legends

**Supplementary Table Legends**

**Supplementary Table S1**: Physical and chemical properties of the deep ocean samples 3 from the South Indian Ocean sampling sites.

**Supplementary Table S2**: Output summary of results from CheckV. The table indicates the quality scores for viruses predicted using the VirFinder pipeline.

**Supplementary Table S3**: Output summary of results from CheckV. The table shows the quality estimates of the putative viruses predicted using the VirSorter2 pipeline.

**Supplementary Table S4**: A table indicating the IDs of various KOs associated with the 85 putative AMGs detected in dsDNA viruses from the SIO.

**Supplementary Table S5**: A list of pathways predicted to be associated with the KOs and putative AMGs detected in dsDNA viruses from the SIO.

**Supplementary Table S6**: Blastp verification of putative ssDNA viruses predicted using VirSorter2 pipeline.

**Supplementary Table S7**: Blastp and HHpred verification of putative Cressdnaviricota-associated Rep proteins predicted using our HMM-based approach.

**Supplementary Table S7**: Blastp verification of putative Phixviricota-associated VP1 proteins predicted using our HMM-based approach.

**Supplementary Figure legends**

**Supplementary Figure S1** Summary statistics for our metagenomic data. The curves together with the dashed red lines show that our metagenomes had estimated coverage >95%.

**Supplementary Figure S2** Bar plots indicating the distribution of Eukaryota associated with the metagenomic dataset.

**Supplementary Figure S3** Sequence similarity networks generated using 1e-60 showing Major Capsid protein sequences acquired from this study and NCBI GenBank.

**Supplementary Figure S4** Sequence similarity networks of genes acquired from “dark matter” associated circular genetic elements (viral) contigs. Clusters are assigned colours based on structural predictions derived from HHpred. Clusters with >85% probability scores are indicated by different colors that distinguishes them from hypothetical proteins.
